# Microbial Succession in the Cheese Ripening Process—Competition of the Starter Cultures and the Microbiota of the Cheese Plant Environment

**DOI:** 10.3390/microorganisms11071735

**Published:** 2023-07-01

**Authors:** Kristyna Korena, Miroslava Krzyzankova, Martina Florianova, Daniela Karasova, Vladimir Babak, Nicol Strakova, Helena Juricova

**Affiliations:** Veterinary Research Institute, Hudcova 296/70, 621 00 Brno, Czech Republic; kristyna.korena@vri.cz (K.K.); miroslava.krzyzankova@vri.cz (M.K.); martina.florianova@vri.cz (M.F.); daniela.karasova@vri.cz (D.K.); vladimir.babak@vri.cz (V.B.)

**Keywords:** smear-ripened cheese, cheese microbiota, starter cultures, 16S rRNA gene sequencing

## Abstract

A large variety of cheeses can be produced using different manufacturing processes and various starter or adjunct cultures. In this study, we have described the succession of the microbial population during the commercial production and subsequent ripening of smear-ripened cheese using 16S rRNA gene sequencing. The composition of the microbiota during the first 6 days of production was constant and consisted mainly of LAB (lactic acid bacteria) originating from the starter culture. From day 7, the proportion of LAB decreased as other bacteria from the production environment appeared. From the 14th day of production, the relative proportion of LAB decreased further, and at the end of ripening, bacteria from the environment wholly dominated. These adventitious microbiota included *Psychrobacter*, *Pseudoalteromonas haloplanktis*/*hodoensis*, *Vibrio toranzoniae*, and *Vibrio litoralis* (Proteobacteria phylum), as well as *Vagococcus* and *Marinilactibacillus* (Firmicutes phylum), *Psychrilyobacter* (Fusobacteria phylum), and *Malaciobacter marinus* (Campylobacterota phylum), all of which appeared to be characteristic taxa associated with the cheese rind. Subsequent analysis showed that the production and ripening of smear-ripened cheese could be divided into three stages, and that the microbiota compositions of samples from the first week of production, the second week of production, and supermarket shelf life all differed.

## 1. Introduction

Cheese is a popular fermented dairy product, the production of which can be accomplished through a variety of production processes and technologies. Specific types of fermented cheeses differ in aroma, taste, texture, or color, and also differ in their microbial composition.

The basic ingredients for cheese production include milk, starter cultures (SCs), adjunct cultures, and salt. In commercial cheese production, milk is pasteurized to prevent the growth of pathogenic bacteria and thus to ensure the microbial safety of the product. Microbial safety is further enhanced by the addition of SCs, mainly lactic acid bacteria (LAB). Their main function is fermentation, i.e., the conversion of available sugars into lactic acid. The desirable side effect of this enzymatic process is the acidification of the product, and thus the formation of an additional barrier inhibiting spoilage flora and pathogens [1,2]. In the next step, yeast cultures are often applied to the cheese to raise the pH by metabolizing lactate on the cheese surface, thereby promoting the growth of adjunct cultures and other acid-sensitive microbiota [3,4]. Optionally, various adjunct cultures, such as non-starter LAB, actinobacteria, yeasts, or molds are deliberately inoculated to influence and control the ripening process and promote biochemical reactions leading to the development of the desired characteristics of the final product.

Cheese can be regarded as a complex ecosystem populated by a diverse group of microorganisms that reflect a particular cheese matrix and substantially interact. Previously, the composition of the cheese microflora was thought to be dominated by bacteria of SCs and/or of adjunct cultures, but it has since been shown that other adventitious microorganisms are present in even greater numbers [5,6]. Bacteria present in the cheese plant environment, which are not usually the primary focus of studies, significantly contribute to the composition of the microflora, and thus to the taste, flavor, and color of the final product.

Sea salt, which is used for the brine solution, is considered as one of the presumed sources of secondary cheese microflora. The so-called marine bacteria, which have been previously detected in marine environments, are reported to be widespread in cheese microbial communities, and have been assumed to be introduced to cheese through the salting step [7,8,9]. These microorganisms, which are now adapted to the cheese plant environment, are halophilic and psychrotolerant in nature. Among them, *Marinomonas*, *Pseudomonas*, and *Psychrobacter* (phylum Proteobacteria) are strongly associated with the production of volatile compounds during cheese ripening. Various kinds of cheeses are also colonized by halophilic lactic acid bacteria, such as *Marinilactibacillus*, *Alkalibacterium*, and *Vagococcus* (phylum Firmicutes). Another frequently detected bacterium is *Pseudoalteromonas* (phylum Proteobacteria), which possesses cold-adapted enzymes, which may contribute to the development of the cheese’s flavor during ripening and storage at low temperatures [10]. Actinobacteria, namely *Brevibacterium* and *Glutamicibacter*, are frequently detected, especially in smear-ripened cheese. These bacteria are considered to be the main producers of sulfur compounds [11], contributing to the typical tangy to strong flavor of red smear cheese, and therefore can be used as adjunct cultures. *Brevibacterium linens* is also responsible for the formation of the orange color that is typically observed on the surface of this type of cheese.

High-throughput sequencing technologies have been used to analyze the microbial communities of different types of ripened cheeses. In general, studies have focused on traditional cheeses made from unpasteurized milk by adding traditional SCs with undefined compositions [12,13]. Other studies have detailed the microbial composition and volatile compound production in long-ripening cheeses [11,14]. However, there are limited studies on microbiota shifts during commercial cheese processing and ripening [1]. The aim of this study was to investigate the succession of microbial populations in smear-ripened cheese, and to reveal the contribution of different adventitious microorganisms present in the cheese plant environment on the composition of the final product.

## 2. Materials and Methods

### 2.1. Smear-Ripened Cheese Production

**Figure 1:**
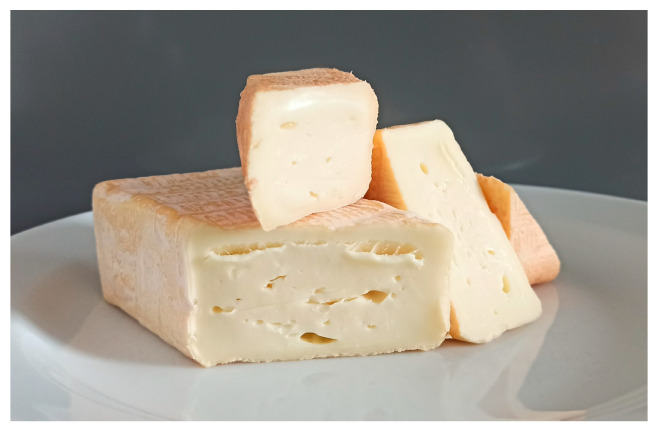
Sample of smear-ripened cheese.

Three batches of smear-ripened cheese (Figure 1) were sampled and examined during production in the cheese processing plant and post-production ripening. Day 1 of the production process included pasteurization of raw cow milk, inoculation with a commercial SC (mixture of *Lactococcus lactis* subsp. *cremoris*, *Lactococcus lactis* subsp. *lactis* biovar *diacetylactis*, *Lactococcus lactis* subsp. *lactis*, and *Leuconostoc*), coagulation with calcium chloride, and formation of the coagulated curd. On the second day of production, the blocks of cheese curd were salted and treated with a yeast culture (*Debaryomyces hansenii*). In the following 2 weeks, the cheeses were treated five times with bacterial adjunct culture *Brevibacterium linens*. During this time, cheeses were stored at 15 °C. On day 15, the final products were wrapped and dispatched to the market. In order to monitor the post-production ripening, the dispatched cheeses were moved to the laboratory and stored at 4 °C until the expiration day.

### 2.2. Sample Collection and Processing

**Figure 2:**
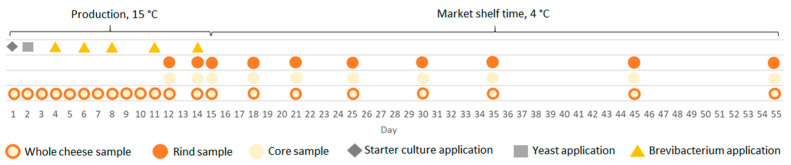
Timing of the whole cheese, rind, and core sampling and depiction of yeast and *Brevibacterium linens* application.

The samples of cheese were taken daily from day 1 to day 12, and then on days 14, 15, 18, 21, 25, 30, 35, 45, and 55 from the production day, respectively (Figure 2). Three individual batches from consecutive production days were then examined. Individual samples of cheese taken on the respective days from day 1 to day 11 (when the core and rind are not yet differentiated), respectively, were processed as a whole. Individual samples of cheese taken on the respective days from day 12 to day 55 were processed as a whole and, in addition, were split into the core and the rind, which were processed separately (Figure 2). Briefly, a total of 25 g of whole cheese, core, or rind sample were homogenized with 225 mL of PBS (phosphate buffered saline) supplemented with 0.2% (*v*/*w*) Tween 80 (PBS-T). Subsequently, 10 mL of the suspension was spun (12,000 g; 30 min; 4 °C) and washed with PBS. The pellet was then subjected to DNA extraction.

Additional three samples were taken from the pasteurized milk prepared for the respective batch of the production. In this case, 15 mL of milk sample was spun and washed with PBS-T, and the resulting pellet was taken for DNA extraction.

### 2.3. DNA Extraction and Microbiota Composition Determined with 16S rRNA Gene Sequencing

DNA was extracted using the DNeasy PowerFood kit according to the manufacturer’s instructions (Qiagen, Germany), with an initial step of homogenization in a MagNALyzer (Roche, Switzerland). The DNA concentration was determined spectrophotometrically and fluorometrically, and DNA samples diluted to 5 ng/mL were used as a template in PCR with the eubacterial primers 5′-TCGTCGGCAGCGTCAGATGTGTATAAGAGACAG-MID-GT-CCTACGGGNGGCWGCAG-3′ and 5′-GTCTCGTGGGCTCGGAGATGTGTATAAGAGACAG-MID-GT-GACTACHVGGGTATCTAATCC-3′, respectively, amplifying the V3/V4 variable region of 16S rRNA genes. Following amplification, the products were processed exactly as described previously [15]. Sequencing was performed using the MiSeq Reagent Kit v3 (600 cycle) and MiSeq apparatus according to the manufacturer’s instructions (Illumina, CA, USA). Raw reads were processed previously described [15].

### 2.4. Data Analysis and Statistics

Downstream analyses on the OTU (operational taxonomic unit) level were performed with OTUs that accounted for more than 0.1% of the total microbiota in at least one sample. These filtered OTUs covered 99.87–100.00% of the total microbiota of the cheese samples and 88.94–92.98% of the total microbiota of milk samples, respectively. Permutational analysis of variance in repeated measures design (RM PERMANOVA) on the Bray–Curtis matrix distances (R-project, package vegan, function Adonis), followed by pairwise comparisons was used to determine the groups (e.g., batches, time stages of the microbiota succession, production days) which significantly differed in terms of their microbial composition. *p*-values of pairwise comparisons were adjusted using the Bonferroni correction. *p*-values lower than 0.05 were considered as statistically significant. Cluster analyses with complete linkage and Bray–Curtis distance matrixes (STATISTICA 13.2; Dell, Inc., Tulsa, OK, USA) were used to determine the time stages of the microbiota succession (W1–6, W7–12, and W14–55 for samples of whole cheeses, respectively; C11–30 and C35–C55 for core samples; and R11–55 for rind samples). Principal coordinate analysis (PCoA) followed by PERMANOVA on the Bray–Curtis distance matrix of groups (W1–6, W7–12, W14–55, C11–30, C35–55 and R11–55) was performed by using package vegan (R-project). The linear discriminant analysis effect size (LEfSe; https://huttenhower.sph.harvard.edu/galaxy (accessed on 2 May 2023)) [16] was used to determine the taxa that most likely explained the differences between the core and the rind samples. The calculation was made with the following parameters: a = 0.05 (alpha value for the Kruskal–Wallis test) and LDA = 2.0 (threshold on the logarithmic LDA score for linear discriminant analysis).

### 2.5. Total Bacteria Count Determination

To estimate the total bacteria count, 10 g of cheese was homogenized with 90 mL of PBS and serial dilutions were prepared. Of the prepared dilutions, 100 µL were plated on PCA agar (plate count agar) supplemented with 5% sodium chloride. Colony-forming units (CFU) were determined after the dilution of the samples with the dilution factor 2. The experiments were performed in three repetitions.

## 3. Results

### 3.1. Microbiota Composition of Milk and Smear-Ripened Cheeses Determined with 16S rRNA Gene Sequencing

**Figure 3:**
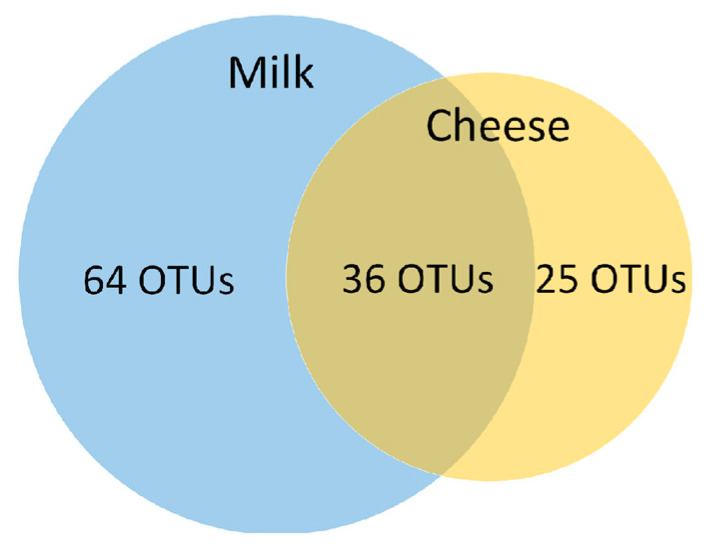
Number of OTUs identified in the milk samples and in the samples of smear-ripened cheese.

The microbiota composition was determined in three production batches of cheese, each comprising one milk sample and 41 cheese samples, respectively. In total, 125 OTUs were detected in all samples. Out of these, 100 and 61 OTUs were detected in the milk and cheese samples, respectively, and a total of 36 OTUs were present in both commodities (Figure 3).

**Figure 4:**
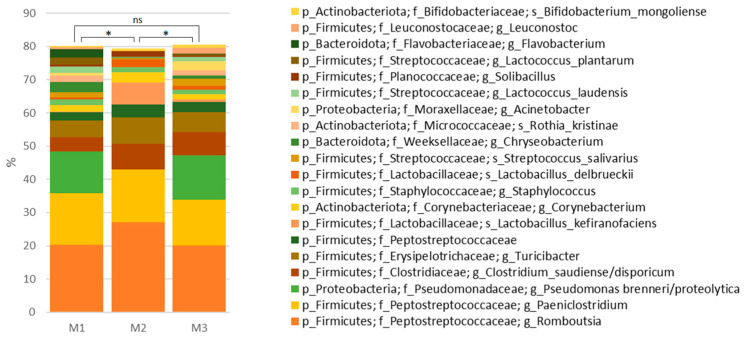
Relative abundances of the 20 taxa with the highest average abundance at the genus and species level in three milk samples prepared for the respective batch of the production of smear-ripened cheese. * *p* < 0.05; ns, not significant.

The microbiota compositions of the two milk samples (M1, M3) prepared for the production of the two batches of cheese were not found to be significantly different (*p* > 0.05; RM PERMANOVA, pairwise comparisons; Figure 4). However, the microflora of the remaining milk sample (M2) did significantly differ (*p* < 0.05; RM PERMANOVA, pairwise comparisons). The most abundant bacterial taxa detected in the milk samples included *Romboutsia*, *Paeniclostridium*, *Pseudomonas brenneri/proteolytica*, *Clostridium saudiense/disporicum*, *Turicibacter*, undefined *Peptostreptococcaceae*, *Lactobacillus kefiranofaciens*, *Corynebacterium*, *Staphylococcus*, and *Lactobacillus delbrueckii* (Figure 4). None of these taxa appeared later in cheese at an abundance higher than 0.1% of the total microbiota.

**Figure 5:**
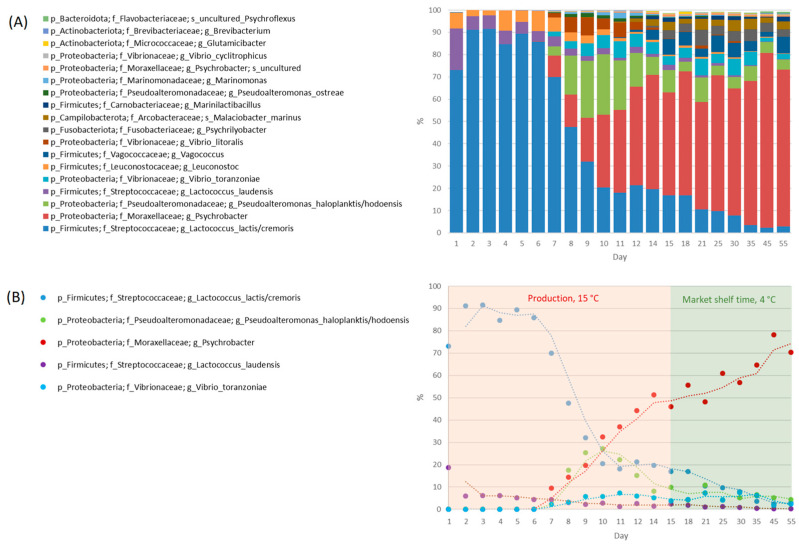
Relative abundances of the 18 taxa with the highest average abundance at the genus and species level in smear-ripened cheese samples. Three production batches were monitored. The microbiota composition in the cheese samples collected on the same day did not differ significantly within the production batches, and data from the respective days were averaged between the batches (**A**). The succession of the five taxa with the highest abundance during production and post-production ripening. Days 1–15, production; and days 15–55, market shelf time. The X-axis is not in a linear time scale (**B**).

The microbiota compositions in cheese samples collected on the same day did not differ significantly between the production batches (*p* > 0.05; RM PERMANOVA), thus allowing data from the respective days to be averaged between batches. The most abundant bacterial taxa detected in cheese samples included *Lactococcus lactis*/*cremoris*, *Psychrobacter*, *Pseudoalteromonas haloplanktis*/*hodoensis*, *Lactococcus laudensis*, *Vibrio toranzoniae*, *Leuconostoc*, *Vagococcus*, *Vibrio litoralis*, *Psychrilyobacter*, and *Malaciobacter marinus* (Figure 5), whose abundance changed considerably during the production and through ripening (see Section 3.2). With the exception of *Lactococcus laudensis* and *Leuconostoc*, the abundance of these taxa did not exceed 0.5% of the total microbiota in the milk samples.

By cultivation, the total number of bacteria in cheese samples was approximately 2.3 × 10^7^ CFUs per gram.

### 3.2. Microbiota Succession during Cheese Production and Ripening

Microbial succession evolved during the cheese production and subsequent ripening. The microbiota composition during the first 6 days was quite constant, and the three most abundant OTUs belonging to the LAB (*Lactococcus* and *Leuconostoc*) accounted for 98.9–99.9% of the total microbiota (Figure 5). On day 7, the proportion of these three taxa decreased to 80.8% as other taxa began to appear. In the following days, the relative abundance of LAB decreased further until it reached a minimum of less than 3% at the end of ripening. In contrast, the relative abundance of the second dominant taxon *Psychrobacter* increased significantly from day 7, reaching a maximum abundance of 78.2% of the total microbiota on day 45. The third most abundant taxon, *Pseudoalteromonas haloplanktis*/*hodoensis,* became abundant during the second week of the production process, accounting for 15.2–27.2% of the total microbiota. Other adventitious bacteria included *Vibrio toranzoniae* and *Vibrio litoralis*, which both increased during the second week of production, and *Vagococcus*, *Psychrilyobacter*, and *Malaciobacter*, which appeared from the third week onwards and maintained a high abundance until the end of ripening.

### 3.3. Differences in the Microbiota Composition of the Core and the Rind

**Figure 6:**
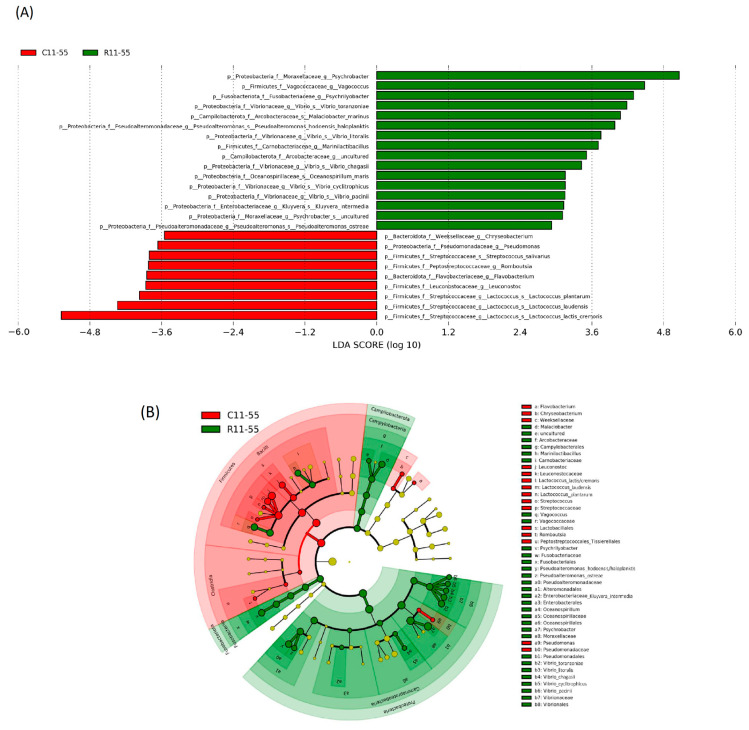
The effect size of particular taxa characteristics for the rind (green) and the core (red) microbiota determined using the LDA effect size (LEfSe) algorithm (**A**). The cladogram was obtained from LEfSe analysis. The particular taxa are highlighted by small circles and by shading (**B**).

From day 12 onwards, the cheese began to form a rind. The duplicate cheese samples collected on days 12 onwards were therefore split into the core and the rind, which were processed and analyzed separately. There were 49 and 36 OTUs detected in the core and the rind, respectively, and all 36 OTUs detected in the rind were also present in the core. LEfSe analysis (LDA, linear discriminant analysis) identified nine characterizing taxa that were found to be specifically associated with the cheese core (Figure 6A). These included three dominant taxa, *Lactococcus lactis*/*cremoris*, *Lactococcus laudensis*, and *Leuconostoc*, all belonging to the LAB of the Firmicutes phylum (Figure 6B). Their abundances in the cheese core were at least an order of magnitude higher than in the cheese rind (differences in log LDA score were greater than 2.0). The remaining six bacterial taxa represented minority taxa whose average abundance did not exceed 0.01% of the total microbiota, but unexpectedly belonged to abundant taxa detected in the milk samples. Altogether, 16 bacterial taxa were revealed as specific to the cheese rind, the most abundant of which were *Psychrobacter*, *Vibrio toranzoniae*, *Pseudoalteromonas haloplanktis/hodoensis*, and *Vibrio litoralis* belonging to Proteobacteria, *Vagococcus* and *Marinilactibacillus* from Firmicutes, *Psychrilyobacter* from Fusobacteria, and *Malaciobacter marinus* belonging to Campylobacterota, respectively (Figure 6A). The remaining eight bacterial taxa represented minority taxa with an average abundance of 0.02–0.55% of the total microbiota and a majority (seven out of eight) belonged to Proteobacteria (Figure 6B).

### 3.4. Clustering of the Microbiota Composition of the Whole Cheese, Core, and Rind

**Figure 7:**
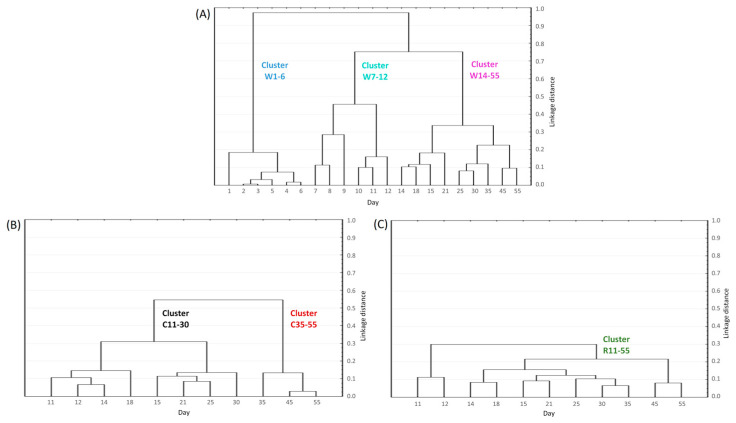
Clustering of the microbiota composition of 63 samples of the whole smear-ripened cheese (**A**), 30 samples of the cheese core (**B**), and 30 samples of the cheese rind (**C**). Three, two, and one major clusters with significantly different microbiota compositions are defined in the whole cheese, core, and rind samples, respectively.

Cluster analysis of microbiota abundance in the whole cheese, core, and rind was performed to reveal major shifts in the succession of the microbiota composition. Three main clusters were defined in the case of the whole cheese samples, indicating that the production and ripening of smear-ripened cheese can be divided into three stages. The microbiota compositions of samples from the first week of production, the second week of production, and supermarket shelf life were all found to be statistically significantly different from one another (*p* < 0.05; RM PERMANOVA, pairwise comparisons; Figure 7A). Two significantly different clusters were defined in the case of the samples of the cheese core. These comprised core samples of days 11–30 and days 35–55, respectively, whose microbiota compositions were found to be statistically significantly different (*p* < 0.05; RM PERMANOVA; Figure 7B). On the other hand, the microbiota composition of all the samples of the cheese rind were not found to be statistically significantly different over the entire ripening period (days 11–55) (*p* > 0.05; RM PERMANOVA, pairwise comparisons; Figure 7C).

**Figure 8:**
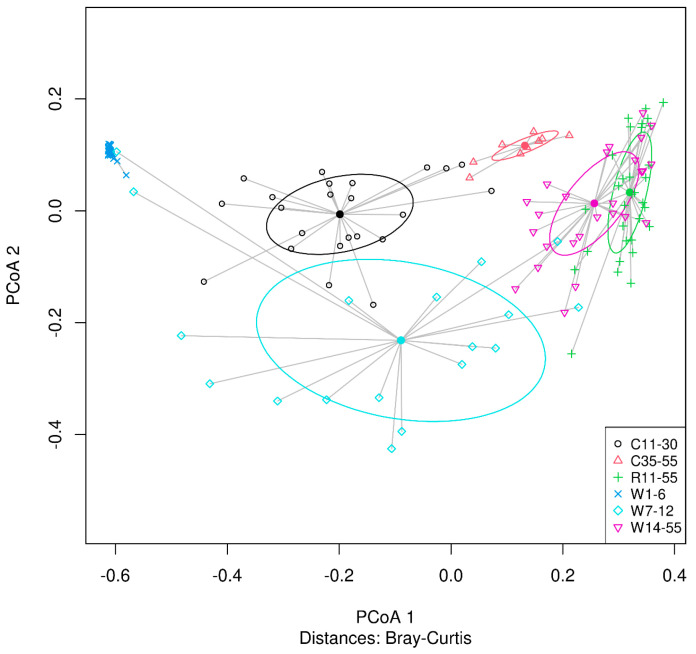
Clustering of the microbial community of 123 samples of smear–ripened cheese based on principal coordinate analysis plot. Clusters—W1–6, samples of whole cheese of days 1–6; W7–12, whole cheese of days 7–12; W14–55, whole cheese of days 14–55; C11–30, samples of cheese core of days 11–30; C35–55, core of days 35–55; R11–55, samples of cheese rind of days 11–55.

PCoA analysis followed by PERMANOVA confirmed that there were statistically significant differences (*p* < 0.05; PERMANOVA, pairwise comparisons) between the composition of the microflora of the whole cheese and the core in different stages of the cheese production and ripening (Figure 8). However, the composition of the rind microflora was not found to be significantly different (*p* > 0.05; PERMANOVA, pairwise comparisons) from the microflora of the whole cheese during the late stage of ripening (days 14–55, respectively).

## 4. Discussion

In this study, we used 16S rRNA gene amplicon sequencing to investigate in detail the microbiota composition of smear-ripened cheese, and to determine how individual bacterial species and microbial groups change over time within the cheese matrix. The microbiota succession was monitored during the commercial cheese production, which included fermentation and initial ripening at 15 °C, and during post-production ripening, which involved storing the final product in the market at 4 °C for a subsequent period of up to 40 days.

The microbiota composition was determined in three individual production batches of cheese and three corresponding samples of pasteurized milk. The composition of the milk microflora was very diverse, and almost a 1000 ASVs (amplicon sequence variants) were detected (data not shown), but their abundance in the cheese samples did not exceed 0.1% of the total microbiota. The pasteurization of milk generally reduced the overall microbial burden rather than completely sterilize the milk. Therefore, one source of cheese microbiota may be, for example, thermoduric bacteria, which have survived pasteurization, and are frequently found in the microbial community of cheese [17]. Indeed, several studies have shown that cheeses made from pasteurized milk contain a less diverse microbiome than those made from unpasteurized milk [17,18,19]. Our results indicate that milk-derived bacteria did not influence the microbiota composition of this type of cheese. This is also consistent with the fact that even though the milk microflora varied significantly, no significant differences in the compositions of the cheese microbiota were observed between these production batches. This could be caused by different conditions in the milk and the ripening cheese, as well as by the efficient milk pasteurization. These observations also reveal that the production process of the commercially produced cheese is both robust and fully standardized.

The most abundant species detected in the cheese samples were *Leuconostoc* and two *Lactococcus* species originating from starter cultures. Other bacteria detected with an increased abundance included bacterial taxa belonging to the phyla Proteobacteria, Firmicutes, Fusobacteria and Campylobacterota. With the exception of *Lactococcus* and *Leuconostoc*, the abundance of these taxa did not exceed 0.5% of the total microbiota in the milk samples. Although we cannot exclude milk as the source, we propose that these bacteria originated from the cheese production environment.

Different microbial communities are involved in different stages of cheese processing and ripening, providing diverse beneficial functions through either individual metabolism or through complex ecological interactions. As determined with 16S rRNA gene amplicon sequencing, the succession of the microflora was observed during the production and subsequent ripening of the smear-ripened cheese. The microbiota of the cheese during the first week of production consisted exclusively of the LAB of *Leuconostoc* and *Lactococcus*. It has been reported that the bacteria of starter cultures dominate mainly in the initial phase of the fermentation process [5,20], when lactose as a major carbohydrate compound is present in high amounts. Later during the fermentation process, lactose is rapidly utilized, and the population of LAB gradually decreases with further cheese aging [5,20]. Similarly, we observed a decrease in the relative abundance of LAB from the second week of production until the end of ripening.

In parallel with the decrease in the LAB, the relative abundance of particularly the Proteobacteria *Vibrio*, *Pseudoalteromonas*, and *Psychrobacter* began to increase from the second week of production, gradually replacing the LAB of the SC. Gram-negative Proteobacteria are frequently detected in smear cheeses [21,22]. These bacteria are known to possess genes involved in the enzymatic degradation of casein and peptides, leading to the production of free amino acids [21,23,24]. *Pseudoalteromonas* spp., detected in soft-type ripened cheese, have been found to possess cold-adapted enzymes that contribute to the cheese flavor development during ripening and storage at low temperatures [10,25]. In our study, *Psychrobacter* was predominant among the cheese microbiota during the late ripening of the cheese. It has been reported that the artificial inoculation of *Psychrobacter celer* into the cheese microbiota can significantly reduce the bacterial community. On the other hand, a large amount of volatile aroma compounds, especially sulfur compounds, have been detected [21]. 

Finally, from the second week onwards, other adventitious bacteria, e.g., *Malaciobacter* and *Psychrilyobacter*, appeared in the cheese microbiota during ripening at refrigeration temperatures, and maintained a high abundance until the end of ripening. The strains of *Malaciobacter* spp. (previously known as *Arcobacter* spp.) have been previously isolated from cow and water buffalo milk [26], sheep ricotta cheeses [27], and the dairy plant environment [28]. While the so-called marine bacteria, such as *Pseudoalteromonas*, *Psychrobacter*, and *Malaciobacter* are described as the typical microbiota of the soft and semi-hard cheeses [10,29,30,31], the presence of *Psychrilyobacter* has never been reported among the cheese microbiota. This genus of the Fusobacteria phylum has been commonly observed in the marine environments in association with marine invertebrates [32], and its presence has rarely been recorded in environments such as fermented foods [33] or the cheese production environment [34].

Smear-ripened cheese is characterized by its unique taste and the yellow-orange color of the rind, which can be achieved through applying an adjunct culture on the surface of the cheese. The microbiota of the cheese rind and the cheese core was therefore compared. The core of the cheese is characteristic by its reduced oxygen level and a lower pH, both leading to a reduced biodiversity [17,35]. This environment favors the LAB, including those in SCs. In agreement, the core samples were characteristic by the presence of OTUs belonging to LAB, such as facultative anaerobic lactococci. Although these bacteria grow better in the presence of oxygen, they take advantage of the bacterial consortia through lactate dehydrogenase, enabling them to grow and obtain energy under anaerobic conditions. In contrast, the cheese rind represents an open ecosystem that is exposed to the environment and can be influenced by many biotic and abiotic conditions. Therefore, aerobic and halophilic bacteria are frequently found on these cheese surfaces [17]. Altogether, 16 bacterial taxa specific to the cheese rind were detected on the surface of the smear-ripened cheese. These included marine bacteria and other halophiles from Proteobacteria (*Psychrobacter*, *Pseudoalteromonas*, and *Vibrio*), Firmicutes (*Vagococcus*, *Marinilactibacillus*), Fusobacteria (*Psychrilyobacter*), and Campylobacterota (*Malaciobacter*), respectively.

Cheese represents a complex ecosystem in which cooperative and/or competitive microbial interactions can occur. In addition to milk, one source of unintended microorganisms residing in dairy products is the production environment. Microorganisms which inhabit processing surfaces and equipment appear to be plant- or product-associated and may be involved in the development of the characteristic taste, flavor, and appearance of a particular dairy product.

## 5. Conclusions

The major shifts in the succession of the microbiota composition were defined during the production and ripening of smear-ripened cheese. During the first two weeks of ripening, which coincide with the time the cheese is in the cheese processing plant, the most significant changes in the composition of the microbiota occurred though the ripening process, which continued for an additional 40 days on the shelves. In addition to the LAB originating from the starter culture, Gram-negative bacterial species of Proteobacteria, Campylobacterota, and Fusobacteria were identified, which may play an essential role in ripening and thus contribute to the organoleptic characteristics of the cheese.

## Data Availability

Data is contained within the article.
